# An Investigation of Age-related Neuropathophysiology in Autism Spectrum Disorder Using Fixel-based Analysis of Corpus Callosum White Matter Micro- and Macrostructure

**DOI:** 10.1007/s10803-023-05980-1

**Published:** 2023-04-20

**Authors:** Melissa Kirkovski, Mervyn Singh, Thijs Dhollander, Ian Fuelscher, Christian Hyde, Natalia Albein-Urios, Peter H Donaldson, Peter G Enticott

**Affiliations:** 1 Cognitive Neuroscience Unit, School of Psychology, Deakin University, Geelong, VIC Australia; 2 Institute for Health and Sport, Victoria University, Melbourne, VIC Australia; 3 Murdoch Children’s Research Institute, Melbourne, VIC Australia

**Keywords:** Autism, Corpus callosum, White matter, Macrostructure, Microstructure, Diffusion MRI

## Abstract

Fixel-based analysis was used to probe age-related changes in white matter micro- and macrostructure of the corpus callosum between participants with (N = 54) and without (N = 50) autism spectrum disorder (ASD). Data were obtained from the Autism Brain Imaging Data Exchange-II (ABIDE-II). Compared to age-matched controls, young adolescents with ASD (11.19 ± 7.54 years) showed reduced macroscopic fiber cross-section (logFC) and combined fiber-density and cross-section (FDC). Reduced fiber-density (FD) and FDC was noted in a marginally older (13.87 ± 3.15 years) ASD cohort. Among the oldest ASD cohort (17.07 ± 3.56 years), a non-significant trend indicative of reduced FD was noted. White matter aberration appears greatest and most widespread among younger ASD cohorts. This supports the suggestion that some early neuropathophysiological indicators in ASD may dissipate with age.

**Supplementary Information:**

The online version contains supplementary material available at 10.1007/s10803-023-05980-1.

Autism spectrum disorder (ASD) is an etiologically complex, pervasive, and life-long neurodevelopmental condition characterized by social impairments and the presence of restricted and repetitive patterns of behavior and interests (RRBI) (American Psychiatric Association, 2013). There is strong evidence for structural and functional neuropathophysiology underlying ASD symptomatology (Girault & Piven, 2020; Philip et al., 2012; Sato & Uono, 2019; Wass, 2011).

The corpus callosum (CC) is the largest commissural tract in the brain and is one of the most commonly implicated white matter (WM) tracts in research investigating the structural neuropathophysiology of ASD. The CC has a crucial role in interhemispheric signal transfer, or connectivity, and tracts between the frontal, parietal, temporal, and occipital lobes, both cortically and subcortically, project through the CC (De Lacoste et al., 1985; Hofer & Frahm, 2006; van der Knaap & van der Ham, 2011). Importantly, aberrant, often reduced, interhemispheric connectivity is most commonly reported in ASD (Anderson et al., 2011; Dickinson et al., 2018; Guo et al., 2020; Li et al., 2019; Zhu et al., 2014). Morphologically, it is understood that CC volume and thickness are reduced in ASD groups compared to controls (Anderson et al., 2011; Frazier et al., 2012; Freitag et al., 2009; Hardan et al., 2009; Li et al., 2019; Temur et al., 2019), though evidence is mixed (Kucharsky Hiess et al., 2015; Lefebvre et al., 2015). Diffusion MRI (dMRI) allows for a deeper understanding of the WM micro- and macrostructural effects that might contribute to these reductions in ASD. Early dMRI based research generally indicates that fractional anisotropy (FA) is reduced in ASD compared to controls. Mean- (MD), axial- (AD), and radial-diffusivity (RD), in contrast, are generally reported to be increased at the CC of individuals with ASD (Yao et al., 2021). There is, however, a great deal of variability in the literature. Numerous studies have reported opposing findings to those described above, and many report no differences in WM microstructure in ASD compared to neurotypical (NT) cohorts (Aoki et al., 2013; Hoppenbrouwers et al., 2014; Travers et al., 2012; Valenti et al., 2020; Yao et al., 2021).

One factor likely contributing to this heterogeneity is chronological age. Age-related effects in the neurodevelopmental trajectory of ASD are well documented. Broadly, these include early brain overgrowth, abnormal structural development, and altered neural connectivity (Girault & Piven, 2020). The dMRI literature suggests an atypical developmental trajectory of WM in ASD, whereby early WM abnormalities appear to ameliorate with age. More specifically, group differences in classic diffusion tensor imaging (DTI) metrics (FA, AD, MD, and RD) often revert to a level where these indices of WM microstructure become comparable to a non-clinical cohort during adulthood. Moreover, recent work suggests that the pattern, or direction, of microstructural aberration might actually “switch” in ASD (Karahanoğlu et al., 2018). Here, the authors report higher FA and AD in younger (aged 8–15) individuals with ASD compared to a matched NT comparison group, while the opposite pattern was observed for the older (16–25 years) cohort (Karahanoğlu et al., 2018). Longitudinal dMRI research further supports these observations (Andrews et al., 2021; McLaughlin et al., 2018; Travers et al., 2015). Thus, while the developmental profile of WM in ASD is yet to be fully elucidated, there is evidence of age-related alterations.

Focusing on the CC in particular, similar age-related patterns and differences have been reported in ASD. Travers and colleagues (2015) conducted a longitudinal dMRI investigation of CC microstructure, following up 100 males with ASD and 56 male NT controls, aged between 3 and 40 years, at four time points. The authors report several age-by-group interactions across DTI metrics and CC sub-regions. These effects can be described by decreased FA across the entirety of the CC, divided into the genu, body, and splenium, and increased MD at the splenium in individuals < 10 years old. In support of the view that microstructural differences in ASD may dissipate with age, no significant age-related effects were observed in the older (10–20 years, and 20 + years) cohorts (Travers et al., 2015). Alternatively, more recent work from Andrews et al. (2021) supports the ‘switching’ hypothesis. This study followed 125 children with ASD and 69 NT controls aged between 2.5 and 7 years old over 2–3 time points. The authors report that the developmental trajectory of FA at the body and splenium was slower in the ASD cohort, indicating increased FA in younger children with ASD and decreased FA in older children with ASD compared to NT children. At the genu, the developmental trajectory of FA was relatively similar to that of the NT group (Andrews et al., 2021). Given the variability in ages investigated, and that the entire age range investigated by Andrews and colleagues (2021) fits within the “younger” group of the earlier longitudinal study by Travers et al. (2015), the age at which these changes in microstructural development occurs is unclear. It is also important to note that microstructural abnormalities are indeed also reported among some studies investigating adults with ASD (Travers et al., 2012), demonstrating that age is likely one of many possible factors contributing to the observed heterogeneity in WM microstructure in ASD.

While age is a major factor contributing to the heterogenous neuropathophysiology of ASD, some of the heterogeneity observed might be attributable to the previously described DTI-based metrics themselves. These older methods of investigating WM microstructure are limited by their inability to account for multiple-, or crossing-, fibers within a voxel (Farquharson et al., 2013; Jeurissen et al., 2013). This is an important consideration, particularly for the CC, given the multitude of fibers crossing through this structure due to its role as the primary pathway for interhemispheric signal transfer throughout the brain (De Lacoste et al., 1985; van der Knaap & van der Ham, 2011). Alternatively, a fixel-based analysis (FBA) approach allows for individual fiber populations (called “fixels”) to be investigated *within* each single voxel, thereby properly accounting for crossing-fibers (Dhollander et al., 2021; Raffelt et al., 2015). This approach improves the biological specificity through which WM can be assessed via indices such as fiber density (FD; a microstructural measure of intra-axonal volume), fiber cross-section (FC; an index of fiber bundle morphology), and combined fiber density and cross-section (FDC; a combined metric assessing the influence of both micro- and macrostructural changes in WM) (Raffelt et al., 2015). Only two studies have applied an FBA approach to ASD data, both implicating the CC in their findings of WM neuropathophysiology, but neither of which investigated age-related effects (Dimond et al., 2019; Kirkovski et al., 2020). Among a sample aged between 14 and 20 years, Dimond et al. (2019) reported reduced FD in the splenium and genu of the CC in individuals with ASD (n = 25) compared to NT controls (n = 27). Beyond the CC, this study also identified reduced FD at the inferior fronto-occipital fasciculus bilaterally, and the right arcuate and uncinate fasciculi (Dimond et al., 2019). In an older sample aged 19–56 years, only a non-significant trend toward reduced FD at the posterior midbody and isthmus of the CC among the ASD group (n = 25) compared to matched controls (n = 24) was identified (Kirkovski et al., 2020).

Leveraging data accessed from the Autism Brain Imaging Data Exchange-II (ABIDE-II) database, here we apply FBA to investigate age-related micro- and macrostructural neuropathophysiology in ASD. The present study, to our knowledge, is the first to apply the FBA framework in this regard. It was hypothesized that greater WM micro- and macrostructural aberration would be present in younger ASD groups, compared to their matched NT counterparts, and relative to older cohorts.

## Methods

As data available via ABIDE have had all identifiable and protected health information removed, are publicly available, and use of these data poses no foreseeable risk of harm or discomfort to participants, an exemption from ethics review was granted by the Deakin University Human Research Ethics Committee for this project (DU-HREC; Reference: 2020-099).

dMRI and anatomical (T1-weighted) image data were obtained from the ABIDE-II (http://fcon_1000.projects.nitrc.org/indi/abide/abide_II.html) database. Five dMRI datasets are available here. Data from one site, New York University Langone Medical Center: Sample 2, did not include a NT comparison group and was not included in this study. In addition, due to missing gradient encoding information, dMRI data from the Institut Pasteur and Robert Debré Hospital site were also not analyzed here. Therefore, dMRI data from 3 sites were analyzed in the present study. These were: New York University Langone Medical Center, Sample 1 (NYU), San Diego State University (SDSU), and the Trinity Centre for Health Science (TCD). Children were acclimatized to the scanning environment at each site by undergoing a mock MRI session before the live scan. Given technical challenges, and limitations and confounds of analyzing multi-site dMRI data using different acquisition parameters (Zhu et al., 2011), data from each site were analyzed separately. All image processing and analyses were performed via the multi-modal Australian ScienceS Imaging and Visualization Environment (MASSIVE) supercomputing infrastructure (Goscinski et al., 2014).

Due to technical and data quality issues described below, several participants were excluded from analysis. For data included for analysis, participant demographics are detailed, by site, in Table 1.

**Table 1:** Summary of demographics and participant characteristics by site

	NYU		SDSU		TCD	
	ASD	NT	ASD	NT	ASD	NT
n	24	13	23	21	7	16
Age [mean (SD)]	11.19 (7.54)	10.04 (4.40)	13.87 (3.15)	13.85 (2.90)	17.07 (3.56)	16.55 (2.95)
Sex (M:F)	21:3	12:1	18:5	19:2	7:0	16:0
Handedness (R:L:A)	19:2:1^1^	13:0:0	20:3:0	17:1:3	7:0:0	16:0:0
FSIQ [mean (SD)]	103.33 (17.13)	112.62 (14.29)	100.52 (13.41)	102.86 (11.84)	112 (13.67)	120.25 (11)
SRS-Total T [mean (SD)]	71 (15.36)^2^	44.5 (6.11)^3^	-	-	76.14 (7.43)	42.56 (6.95)

*Note*: M = male, F = female, R = right, L = left, A = ambidextrous, FSIQ = full IQ standard score, SRS-Total = Social Responsiveness Scale Total T score; NYU Sample 1: New York University Langone Medical Centre Sample 1; SDSU: San Diego State University; TCD: Trinity Centre for Health Science

^1^ data missing for 2 ASD participants, ^2^ data missing for 3 ASD participants, ^3^ data missing for 1 NT participant, - no data

An overview of the acquisition parameters, as provided to ABIDE-II, used in each scanning site is presented in Table 2. Further site-specific details can be sourced from: http://fcon_1000.projects.nitrc.org/indi/abide/abide_II.html.

**Table 2:** Acquisition parameters per site: dMRI and T1

	NYU		SDSU		TCD	
Manufacturer	Siemens Magnetom		GE M7R50		Philips Achieva	
Tesla strength	3		3		3	
	**dMRI**	**T1**	**dMRI**	**T1**	**dMRI**	**T1**
Sequence	EPI	MPRAGE	EPI	FSPGR	EPI	MPRAGE
Voxel size (mm3)	3 × 3 × 3 × 5.2	1.3 × 1 × 1.3	1.875 × 1.875 × 2 × 1	-	1.938 × 1.938 × 2 × 1	0.89 x 0.89 x 0.89
Slice thickness (mm)	3	1.33	2	1	2	
Orientation	Axial	Sagittal	Axial	Sagittal	Axial	Transverse
Flip angle	60	7	-	8	90	8
b value (s/mm3)	1000	-	1000	-	1500	-
dirs/b = 0	64/1	-	61/1	-	61/1	-
Acquisition time (min: sec)	5:43	8:07	-	-	24:21.7	05:43.6
TE (ms)	78	3.25	Minimum	Min Full	79	shortest
TR (ms)	5200	2530	8500	-	20,244	8.4
FoV (mm)	192 × 192	256 × 256	128 × 128	256 × 256	248 × 248	256 × 256

*Note*: NYU Sample 1: New York University Langone Medical Centre Sample 1; SDSU: San Diego State University; TCD: Trinity Centre for Health Science; TE: Echo time; TR: Repetition time; FoV: Field of view; EPI: Echo Planar Imaging; - no data; MPRAGE: Magnetization-prepared 180 degrees radio-frequency pulses and rapid gradient-echo; FSPGR: Fast spoiled gradient-echo resonance

### dMRI data Preparation

Quality assessment was performed by two raters (MK and MS). All steps were performed in MRtrix3Tissue (v5.2.8. https://3tissue.github.io/), a fork of the MRtrix3 software package (Tournier et al., 2019). Prior to processing, the raw data from each scanning site were assessed to ensure they met basic quality checks. Subjects were removed if they were missing dMRI data (NYU: n = 21; SDSU: n = 1; TCD: n = 2) or gradient encoding information (NYU: n = 2). The data for all remaining subjects were then converted from NIfTI to MRtrix3-native format.

### Quality Assessment

Gradient encoding information for all converted images was checked to ensure that the correct orientations were preserved during the conversion, which led to the removal of one subject where this information was corrupted (SDSU: n = 1). The data were then visually inspected by both raters for evidence of physiological (e.g. motion) or hardware (e.g. indicated by the presence of Venetian blinding and/or signal dropout) artefacts in areas where we would expect the CC to traverse. Where there was a discrepancy between the raters decision regarding data quality and inclusion/exclusion, consensus was made via a joint discussion. Given that the data were not optimized for FBA analysis, we adopted a particularly conservative approach, and any evidence of artefact resulted in data exclusion. This process resulted in the removal of 48 subjects (NYU: n = 18; SDSU: n = 13; TCD: n = 17), refer to Supplementary Table S1. Based on the quality-control inspection, the final sample consisted of 37 subjects for the NYU (ASD = 24; NT = 13), 44 for the SDSU (ASD = 23; NT = 21), and 23 for the TCD (ASD = 7; NT = 16) scanning sites, respectively.

### Head Motion and Intracranial Volume

All three sites provided participants with at least one mock scanning session prior to data collection so as to acclimatize participants to the scanner environment and reduce in-scanner motion during data collection. We derived quantitative estimates of in-scanner head motion and total brain volume for all subjects in our FBA analysis. In-scanner head motion was operationalized as mean frame-wise displacement (FWD) and calculated across each subject’s raw dMRI dataset (excluding the b = 0 volume) in FSL (Jenkinson et al., 2012) using the approach described by Power et al. (2012). Brain volume measures were calculated separately for gray matter (GM), WM and cerebrospinal fluid (CSF) using T1-weighed structural images in FreeSurfer (Fischl, 2012). We also calculated the estimated total intracranial volume (eTIV). Mann-Whitney tests revealed no statistically significant group differences in FWD and brain volume across all scanning sites (see Supplementary Table S2).

### Preprocessing and Fixel-based Analysis

Data were preprocessed according to a state-of-the-art workflow for FBA (Dhollander et al., 2021). We applied the same steps across all subjects for each respective scanning site. Briefly, each dataset underwent denoising (Veraart et al., 2016) and Gibbs unringing (Kellner et al., 2016) before correcting for motion and eddy-current distortions (Andersson & Sotiropoulos, 2016). Tissue-specific response functions for WM, GM and CSF dMRI signals were estimated individually from each subject’s preprocessed image using an unsupervised method (Dhollander et al., 2019). These response functions were then averaged across subjects in each cohort to generate group-level response functions (Dhollander et al., 2021).

Data were then upsampled to a voxel size of 1.5 × 1.5 × 1.5 mm^3^ to improve spatial anatomical contrast and single-shell 3-tissue constrained spherical deconvolution (SS3T-CSD) was conducted to obtain WM Fiber Orientation Directions (FODs) for each subject (Dhollander & Connelly, 2016). We further applied group-level intensity normalization to ensure that FOD amplitudes were directly comparable across subjects within each separate (scanning site) dataset. To ensure the biological plausibility of FODs underlying the CC, we then performed an additional quality control step, where FOD maps for each subject were visually inspected to determine whether they had adequately accounted for crossing fibers (particularly within the distal regions of the CC), and their spatial alignment with the direction of diffusion along the CC. All subjects showed plausible FODs and were therefore included in all analyses.

Subjects in each cohort were then registered to an unbiased population template space using non-rigid registration. Subject details for each template image are as follows: NYU = 37 (ASD = 24; NT = 13), SDSU = 44 (ASD = 23; NT = 21), and TCD = 23 (ASD = 7; NT = 16). All population templates were further warped to Montreal Neurological Institute (MNI) space in FSL (v6.0.1) to compare regions of significance with those of previous work (Dimond et al., 2019; Kirkovski et al., 2020). Further to this, whole-brain fixel analysis masks were estimated for each template by thresholding peak FOD values to 0.1 for all datasets. Lastly, fixel-wise metrics of FD, FC and FDC were computed across all template fixels for subsequent inferential analysis (Raffelt et al., 2017). FC estimates specifically were log-transformed (logFC) before statistical analysis.

### Tractography

We employed a tract-of-interest approach. The CC was delineated in each population template using TractSeg (Wasserthal et al., 2018), an automated method that provides segmentation of 72 major WM tracts learned from a reference dataset of 105 subjects acquired from the Human Connectome Project (Van Essen et al., 2013).

### Connectivity-based Fixel Enhancement

Group differences in FD, FC and FDC for all tracts-of-interest were conducted using threshold-free Connectivity-based Fixel Enhancement (CFE) (Raffelt et al., 2015). We employed the following default parameters: smoothing = 10 mm full width at half maximum, C = 0.5, E = 2, H = 3. For analyses involving logFC and FDC, we further adjusted for estimated total intracranial volume (eTIV) to account for individual differences in head size on our results. Age was also included as a covariate of interest for all analyses, given its established role in the neuropathophysiology of ASD. All analyses were corrected for multiple comparisons using familywise error rates (FWE). Where significant results were identified (*p*^*FWE*^<0.05), tractograms were cropped so that only streamlines traversing through significant fixels were displayed to better ascertain the WM pathways implicated in the neuropathophysiology of ASD.

### Correlations Between Age and Fixel-wise Metrics

Where significant differences in fixel-wise metrics were observed between groups, FD, logFC or FDC values were then extracted (averaged across the CC) and plotted against age using RStudio v.4.0.5. Due to the relatively small sample sizes within each data set, Spearman’s Rank Order Correlations were used to assess the relationship between age and each of the FBA metrics implicated in the neuropathophysiology of ASD. A p-value of < 0.01 was considered statistically significant to account for multiple comparisons, based on a simple Bonferroni correction.

### Code and Data Availability

All analysis scripts used in the present study are publicly available via: https://github.com/MervSingh/ABIDE-II_FBA.git. The ABIDE-II datasets can be downloaded via: https://fcon_1000.projects.nitrc.org/indi/abide/.

## Results

### Comparison of White Matter Micro- and Macrostructure Between Groups

#### Younger Adolescents (NYU site)

**Fig. 1:**
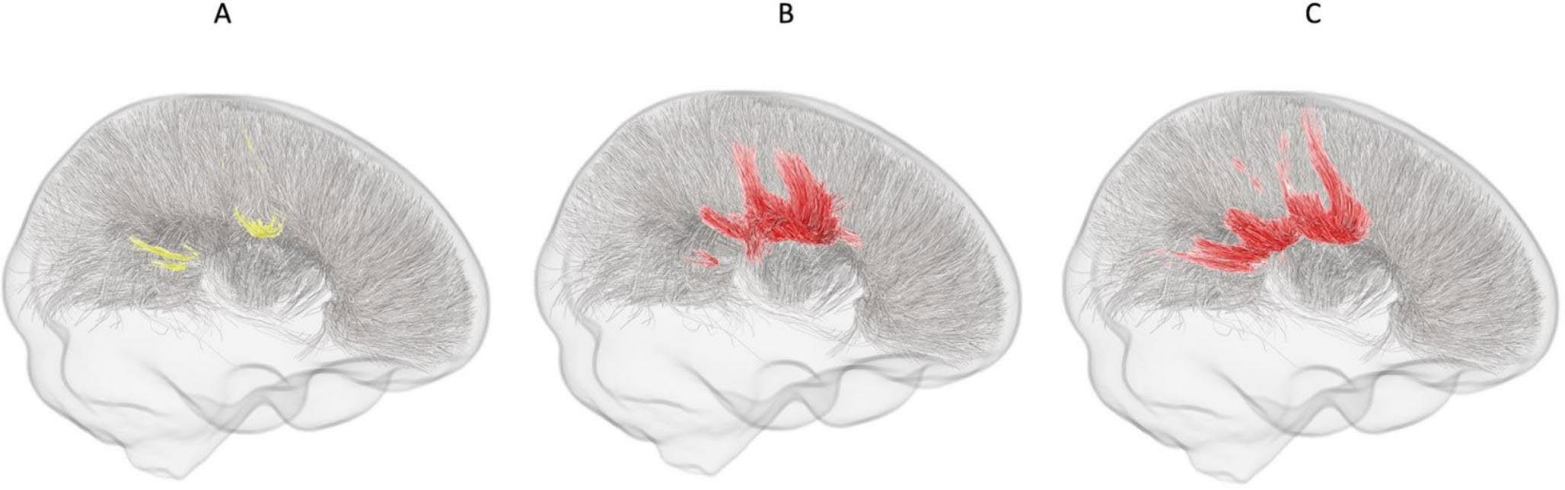
Younger adolescents (NYU site). Graphical representation of streamlines for fixels showing a trend-level reduction in (A) FD, and statistically significant (*p*^FWE^ < 0.05) reductions in (B) logFC, and (C) FDC in the ASD group compared to NT controls. Trend level streamlines are represented in yellow, while significant streamlines are red

FBA revealed significantly decreased logFC and FDC among the ASD group compared to NT controls, particularly around the midbody, isthmus and splenium regions of the CC. A non-significant fixel cluster (*p*^*FWE*^ = < 0.08) indicative of reduced FD among the ASD group was also identified at these regions. Within this cluster, only one fixel remained statistically significant at (*p*^*FWE*^ = < 0.05). Streamline segments of implicated fixels are presented in Fig. 1. No regions with increased FD, logFC, or FDC among the ASD group wereidentified.

#### Older Adolescents (SDSU site)

**Fig. 2:**
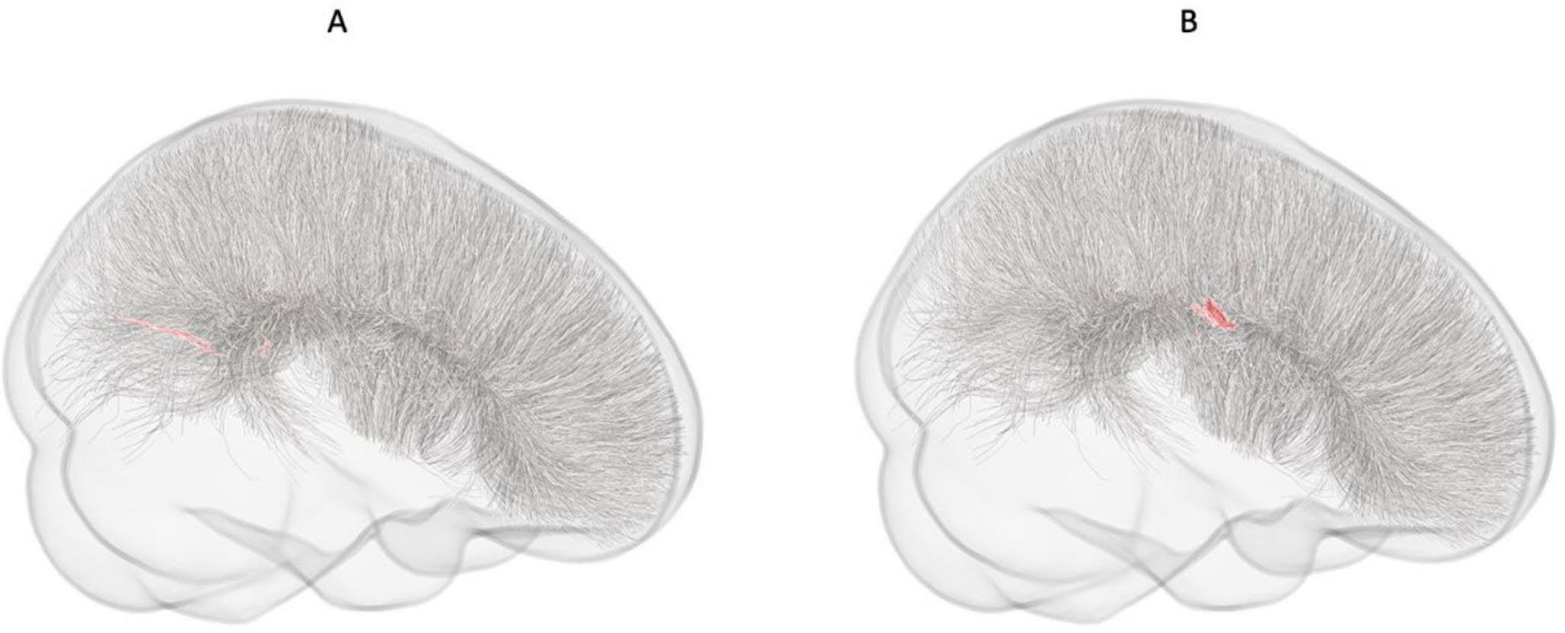
Older adolescents (SDSU site). Graphical representation of streamlines for fixels showing statistically significant (*p*^FWE^ < 0.05) reductions in (A) FD, and (B) FDC in the ASD group compared to NT controls

FBA revealed a statistically significant reduction in FD in the ASD group in a small region of the splenium. A significant reduction in FDC at the midbody was also identified in the ASD group compared to controls. Streamline segments of implicated fixels are presented in Fig. 2. No regions with increased FD, logFC, or FDC among the ASD group were identified.

#### Older adolescents/young Adults (TCD site)

**Fig. 3:**
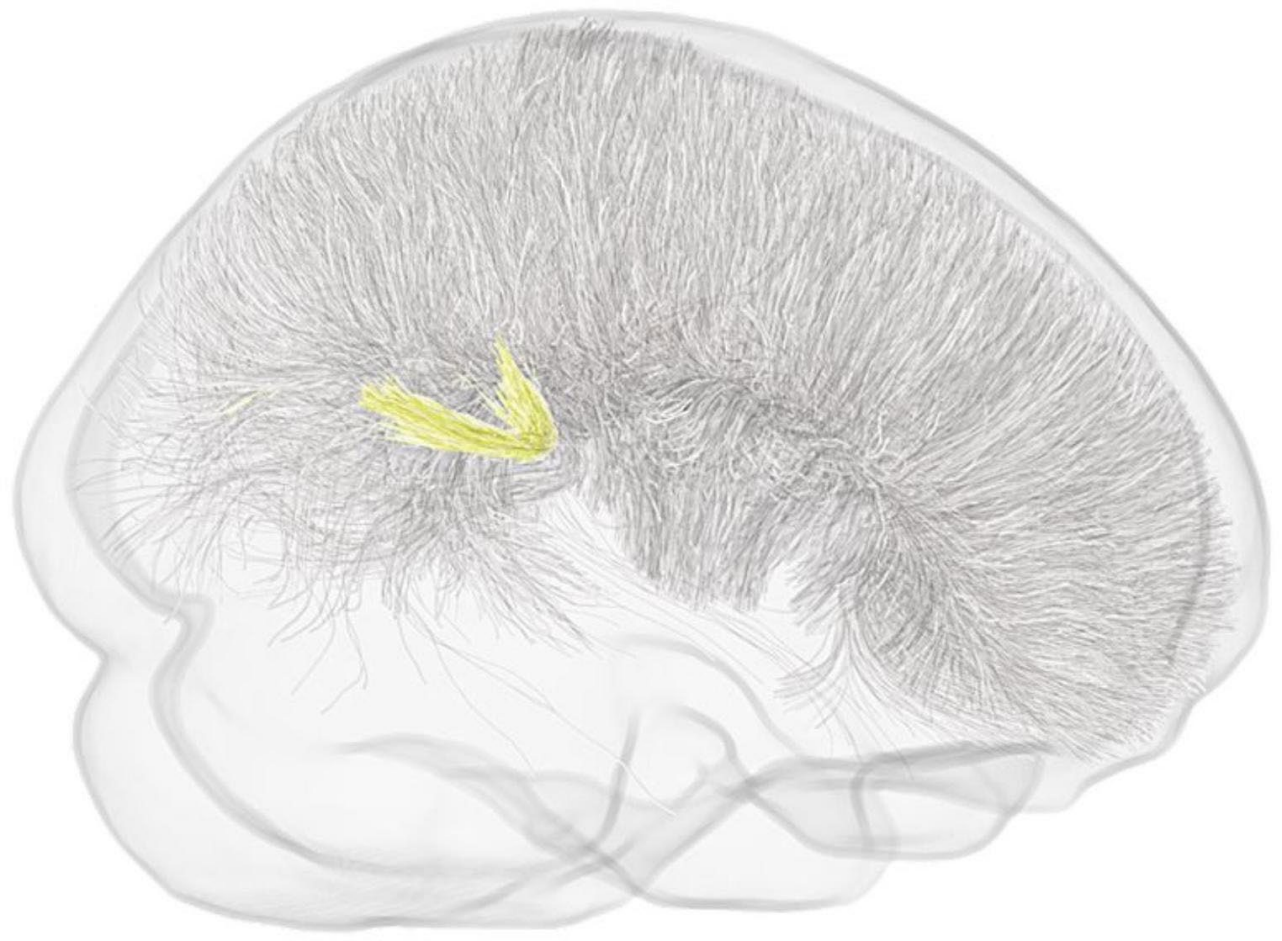
Older adolescents/young adults (TCD site). Graphical representation of streamlines for fixels showing a non-significant trend (*p*^FWE^ = 0.08) toward reductions in FD among the ASD group compared to NT controls. Trend level streamlines are represented in yellow

FBA analysis did not reveal any significant differences in WM micro- or macro-structure between ASD participants and NT controls within this data set. A non-significant trend (*p*^*FWE*^ = 0.08), however, indicative of reduced FD around the isthmus/splenium in the ASD group compared to NT controls was observed (Fig. 3). Within this cluster, only a single fixel remained statistically significant at (*p*^*FWE*^ = < 0.05). No regions with increased FD, logFC, or FDC among the ASD group were identified.

### The Relationship Between Age and White Matter Micro- and Macro-Structure

#### Younger Adolescents (NYU site)

**Fig. 4:**
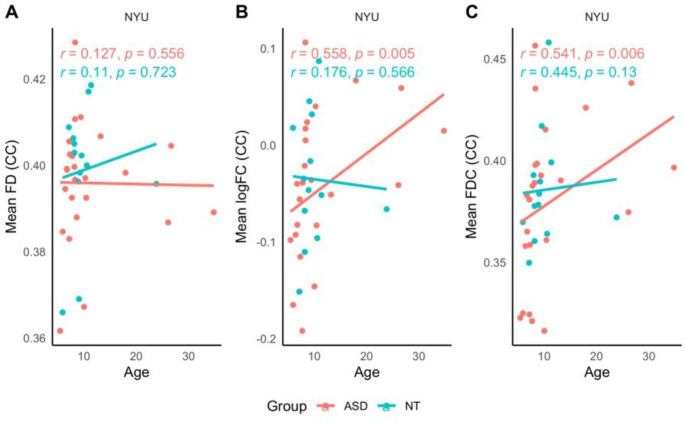
Younger adolescents (NYU site). Scatterplot depicting the relationship between age and mean FD, mean logFC and mean FDC of the CC, stratified by diagnosis

Spearman’s Rank Order Correlations revealed a moderate positive relationship between age and mean logFC (r = .43, *p* = .009) and mean FDC (r = .52, *p* = .001) for the NYU site. When data were stratified by diagnosis, a similar pattern was observed for the ASD group between age and mean logFC (r = .56, *p* = .005), and also age and mean FDC (r = .54, *p* = .006), refer to Fig. 4. There was no relationship between age and either of the relevant fixel-wise metrics for the NT group at this site.

#### Older Adolescents (SDSU site)

**Fig. 5:**
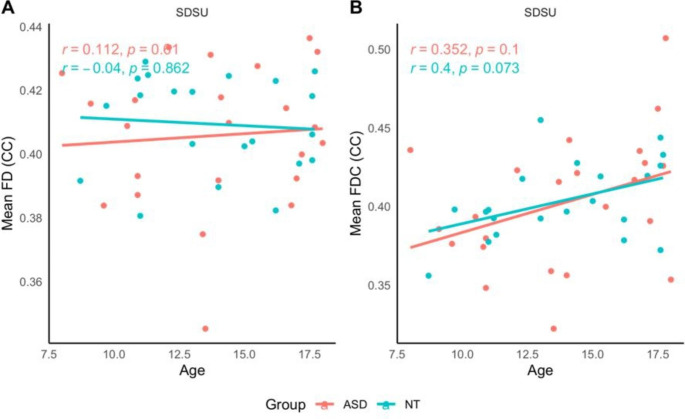
Older adolescents (SDSU site). Scatterplot depicting the relationship between age and mean FD and mean FDC of the CC, stratified by diagnosis

Spearman’s Rank Order Correlations revealed no significant relationships between age and mean FD or mean FDC across the whole sample, nor when data were stratified by diagnosis. Scatter-plots are presented in Fig. 5.

#### Older Adolescents/Young Adults (TCD site)

**Fig. 6:**
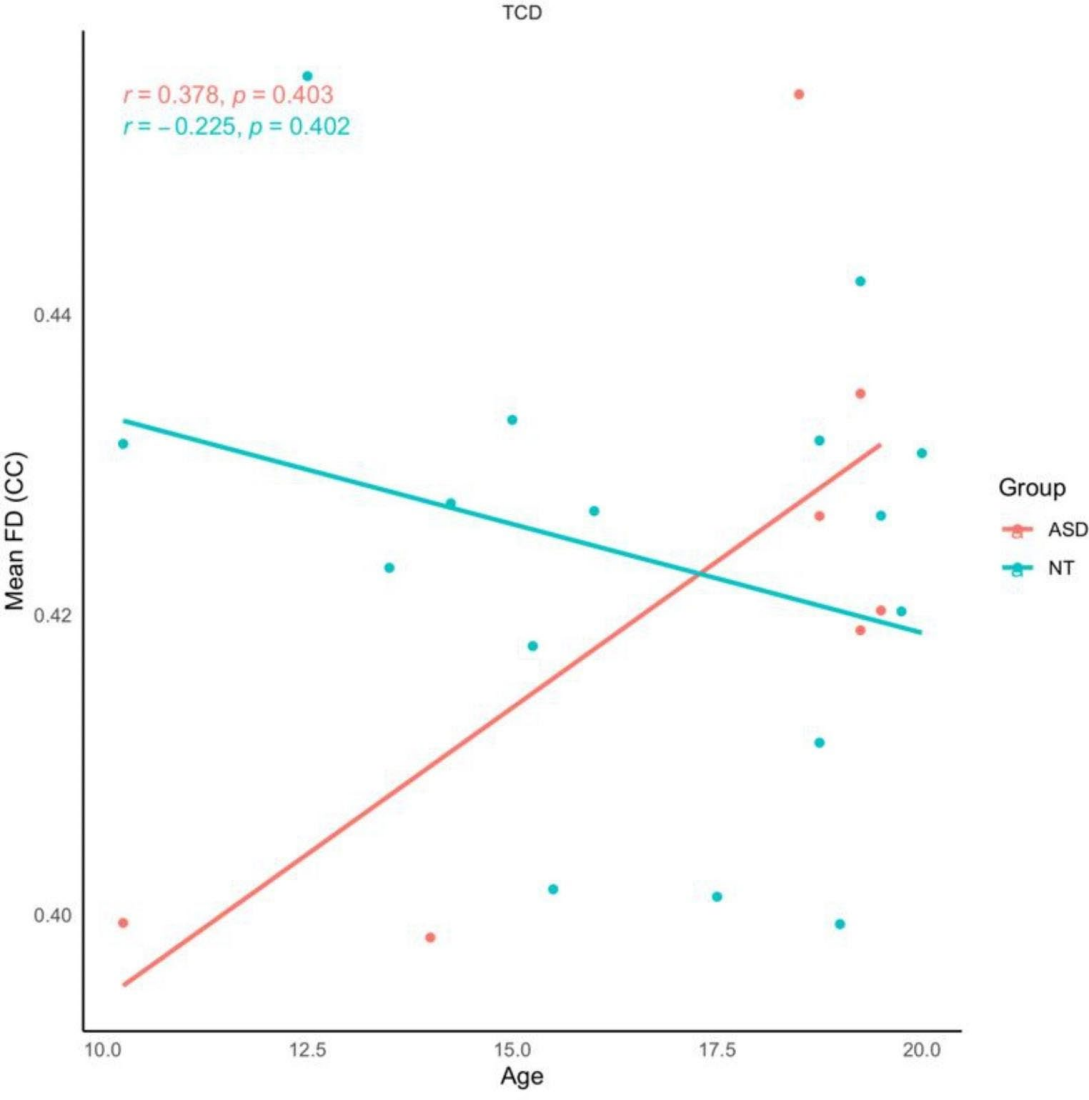
Older adolescents /young adults (TCD site). Scatterplot depicting the relationship between age and mean FD of the CC, stratified by diagnosis

Across the entire TCD dataset, there was no statistically significant relationship between age and mean FD. Similarly, there were no relationships between these variables when data were stratified by diagnosis. Refer to Fig. 6.

## Discussion

We applied fixel-based analysis (FBA) to data from three sites obtained from the ABIDE-II database to investigate age-related WM micro- and macrostructural differences in adolescents and young adults with ASD compared to matched controls. Data for each site are analyzed and reported separately. As hypothesized, the youngest ASD cohort showed the greatest WM aberration compared to their NT counterparts. Differences were identified in micro- and macrostructural measures, implicating the midbody, isthmus and splenium. Less, albeit significant, aberration was identified in overlapping regions in the marginally older cohort. No significant differences in WM micro- or macro-structure were identified among the oldest cohort, though there was a non-significant trend towards reduced FD around the isthmus/splenium region in the ASD group. To reconcile the findings, we provide evidence of altered WM micro- and macrostructure, affecting a greater proportion of the CC in younger, compared to older adolescent and young adult ASD cohorts. Among the older cohort, some aberration was observed in microstructure (FD) only, and was more spatially localized.

Young adolescents with ASD (average age 11.19 [SD = 7.54]) had significantly reduced logFC, and FDC compared to matched NT controls (mean age 10.04 [SD = 4.40]). A non-significant trend indicative of reduced FD in the ASD group was also observed in a small fixel cluster. Statistically significant reductions in FD and FDC were, however, identified in overlapping regions in the slightly older ASD group. As a reminder, reductions in FD might reflect axonal loss or displacement, whereas FC provides a fixel level index of fiber bundle volume relative to the study-specific template (Dhollander et al., 2021; Raffelt et al., 2015, 2017). FDC, however, is arguably a more sensitive (and less specific) measure derived from a combination of FD and FC. As explained by Dhollander et al. (2021), there are several drawbacks to interpreting these metrics alone (i.e., each metric in isolation, without context from the other metrics). Therefore, when considering the results presented here, despite non-significant FD outcome, it is reasonable to conclude that both WM micro- and macrostructure is perturbed in young adolescents with ASD.

Among the older adolescent/young adult cohort, there was trend-level evidence of reduced FD at the isthmus/splenium in the ASD group. This finding is in line with a recent FBA investigation of WM neuropathophysiology in adults with ASD (Kirkovski et al., 2020), where a trend towards reduced FD at the posterior midbody/isthmus was identified among the ASD group. Using diffusion kurtosis imaging (DKI) Sui et al. (2018) provide further evidence aligned with these findings. Specifically, the authors report evidence of reduced axonal density and a reduction in the number of intra-axonal structures at the midbody, isthmus, and splenium of young adults (18–25 years) with ASD compared to matched controls. These findings represent an emerging pattern whereby WM aberration appears concentrated to posterior regions of the CC among older ASD cohorts. In their longitudinal study, Andrews et al. (2021), demonstrate slower development of the splenium of the CC in children with ASD. In considering that several temporal and parietal projections pass through this region of the CC (De Lacoste et al., 1985; Hofer & Frahm, 2006), and the critical role of these cortices in social understanding, a fundamental criterion of ASD (Patriquin et al., 2016), further investigation into the clinical relevance of this finding is warranted.

To better understand the role of age in these findings, correlation analyses for the youngest cohort revealed an age-related increase in these metrics among the ASD group only. No significant relationships were identified among any of the older cohorts. This could be interpreted to reflect findings from early research investigating brain morphology which indicate that a pattern of early brain overgrowth in young children with ASD might likely be followed by a hasty cessation of growth, resulting in similarly sized brains, and brain regions, when comparing older children with ASD to their neurotypical counterparts (Courchesne, 2004; Courchesne et al., 2011; Courchesne & Pierce, 2005). It is important to note, however, that this finding is not universally observed. In a large replication study, Yankowitz et al. (2020) report enlarged brain volume in ASD, from childhood through to adulthood.

Less is understood about the functional overlap with regard to this pattern of age-related stabilization of the brain-growth trajectory in ASD. Using concurrent transcranial magnetic stimulation and electroencephalography (TMS-EEG) Jarczok et al. (2016) report similar maturation effects of interhemispheric signal propagation, indicative of interhemispheric connectivity, in adolescents and young adults with and without ASD. Though these findings might broadly be interpreted to suggest that measures of CC function are not consistent with structural imaging findings, an important consideration here is that interhemispheric signal propagation was measured from the primary motor cortex. In the present study, we observed some degree of comparable WM, primarily around the midbody regions of the CC, fibers of which project into motor, somatosensory and posterior parietal regions (Hofer & Frahm, 2006). While not statistically significant, the present results, and the results of an earlier FBA investigation of WM micro- and macrostructure in ASD (Kirkovski et al., 2020) indicate that some abnormalities might remain at more posterior regions of the CC (isthmus and splenium), projecting in temporal and parietal structures (Hofer & Frahm, 2006), as mentioned above. Indeed, while not investigating the effects of age, Kana et al. (2014) report reduced temporo-parietal FA and blood oxygen level dependent (BOLD) response in ASD during a task measuring social understanding. Taken together, these findings suggest that the structural/functional overlap could be region specific.

Vital to consider is the clinical impact of these age-related changes in the neuropathophysiology of ASD, but research that has systematically and directly investigated this is scarce. Behaviorally, there is evidence to suggest that the severity of ASD symptomatology might abate slightly with age, particularly for those with a less severe clinical profile (Seltzer et al., 2003, 2004; Taylor & Seltzer, 2010), more direct comparison with the neural mechanisms underlying symptomatology is warranted.

### Strengths, Limitations and Future Directions

A key strength of the present study is that FBA, a cutting-edge framework for dMRI analysis, capable of accounting for crossing fibers, has been consistently applied with identical processes, to investigate the role of age on WM pathophysiology in ASD across data from three separate sites. There are, however, important limitations to be acknowledged. Firstly, as described above, combining dMRI data across different acquisition protocols and/or sites to drive more statistically powerful analyses is not straightforward. Even with efforts to harmonize data, this risks introducing biases in the analysis. It might not even be reasonable or sensible to attempt combining dMRI data acquired at, for example, different b-values (diffusion weightings), as such data would be sensitized to fundamentally different aspects of microstructure. Therefore, the data presented here should be considered as three separate studies and interpreted as such. Further to this point, the samples available from each site in our dataset are independently small, particularly for demanding neuroimaging analyses such as FBA. This is a critical consideration, particularly for the TCD sample. Hence, while cautious in our interpretations regarding the non-significant outcomes identified based on these data, they nevertheless support the notion that ASD neuropathophysiology might reduce with age. Another related consideration is that while these datasets have been used to delineate different age groups, separate comparisons limit our ability to account for characteristics inherent to each sample or study design. Finally, regarding the data, we acknowledge the overlap in age across our samples, particularly that the age range of our youngest (average age) sample (NYU) did overlap with the two older sites.

An important methodological consideration is that these data are not entirely optimized for FBA analysis, but had likely been acquired for DTI-based modeling approaches. Higher b-values (e.g., 3000 s/mm^2^) during acquisition yield a better contrast to noise ratio, which promotes better FOD quality from which the FD metric is derived. The FD metric itself is also more specific to intra-axonal volume (which it is meant to represent) at such higher b-values. Though still appropriate for FBA analysis in principle, given the lower b-values of the data presented here we took additional steps to ensure FOD quality (Dhollander et al., 2021), as described in the methods section. Nevertheless, the available data provides important insights into the age-related micro-and macrostructural neuropathophysiology of ASD, setting up the basis for large scale and technically optimized investigation.

Finally, we were limited to the available data. Several factors other than age, such as biological sex (Kirkovski et al., 2013, 2020) clinical features (Giuliano et al., 2018) and symptom severity (Andrews et al., 2021) are known or exceedingly likely to contribute to the heterogeneity observed in ASD and should also be systematically evaluated by future research.

Here we provide evidence that WM aberration is greatest in younger ASD cohorts, and the extent of this aberration is reduced, or altered, in older cohorts. Further investigation of WM pathology in a broader age-range, as well as in larger cohorts, using this method will be of great benefit to the field. Future research should also consider other factors known to contribute to the heterogeneity of ASD, such as biological sex, symptomatology, and cognitive ability.

### Electronic supplementary material

Below is the link to the electronic supplementary material.

Supplementary Material 1
